# Needs of multimorbid heart failure patients and their carers: a qualitative interview study and the creation of personas as a basis for a blended collaborative care intervention

**DOI:** 10.3389/fcvm.2023.1186390

**Published:** 2023-11-10

**Authors:** Petra Engelmann, Natasja Eilerskov, Trine Thilsing, Francesco Bernardini, Sanne Rasmussen, Bernd Löwe, Christoph Herrmann-Lingen, Sara Gostoli, Frida Andréasson, Chiara Rafanelli, Susanne S. Pedersen, Tiny Jaarsma, Sebastian Kohlmann

**Affiliations:** ^1^ Department of Psychosomatic Medicine and Psychotherapy, University Medical Center Hamburg-Eppendorf, Hamburg, Germany; ^2^ Research Unit of General Practice, Institute of Public Health, University of Southern Denmark, Odense C, Denmark; ^3^ Department of Psychology “Renzo Canestrari”, University of Bologna, Bologna, Italy; ^4^ Department of Psychosomatic Medicine and Psychotherapy, University of Göttingen Medical Centre, Göttingen, Germany; ^5^ German Centre for Cardiovascular Research (DZHK), Partner Site Göttingen, Göttingen, Germany; ^6^ Department of Health, Medicine and Caring Sciences (HMV), Linköping University, Linköping, Sweden; ^7^ Department of Psychology, University of Southern Denmark, Odense M, Denmark; ^8^ Department of Cardiology, Odense University Hospital, Odense, Denmark

**Keywords:** heart failure, multimorbidity, blended collaborative care, informal carers, care needs, qualitative study

## Abstract

**Introduction:**

Involving patients and carers in the development of blended collaborative care (BCC) interventions for multimorbid heart failure (HF) patients is recommended but rarely practised, and research on the patient perspective is scarce. The aim of this study is to investigate patients’ and carers' care-related needs and preferences to better customize a novel international BCC intervention.

**Methods:**

A qualitative study design using framework analysis was employed. The study was performed in accordance with the EQUATOR standards for reporting qualitative research (SRQR). Patients aged at least 65 years with HF and at least two other physical diseases as well as their carers completed semistructured interviews in Germany, Italy, and Denmark. Based on these interviews, personas (prototype profiles of patients and carers) were created.

**Results:**

Data from interviews with 25 patients and 17 carers were analysed. Initially, seven country-specific personas were identified, which were iteratively narrowed down to a final set of 3 personas: (a) the one who needs and wants support, (b) the one who has accepted their situation with HF and reaches out when necessary, and (c) the one who feels neglected by the health care system. Carers identifying with the last persona showed high levels of psychological stress and a high need for support.

**Discussion:**

This is the first international qualitative study on patients' and carers' needs regarding a BCC intervention using the creation of personas. Across three European countries, data from interviews were used to develop three contrasting personas. Instead of providing “one size fits all” interventions, the results indicate that BCC interventions should offer different approaches based on the needs of individual patients and carers. The personas will serve as a basis for the development of a novel BCC intervention as part of the EU project ESCAPE (Evaluation of a patient-centred biopSychosocial blended collaborative CAre Pathway for the treatment of multimorbid Elderly patients).

## 1. Introduction

Heart failure (HF) is a growing public health problem that affects more than 60 million people around the globe (1). Despite improvements in care, the mortality of HF has remained substantially unchanged in recent years (2). In addition, multimorbidity is highly prevalent in people suffering from HF (3) and is associated with worse outcomes, including increased health care use, higher costs, and death (4, 5). The treatment of patients with HF and other comorbidities is still challenging (6, 7). While multidisciplinary care management programs are recommended (8), they have yielded mixed results in terms of effectiveness and have failed to impact the combined outcomes of mental and somatic health (9, 10). To improve effectiveness, multidisciplinary programs should be better tailored to the needs of those affected. However, previous studies on intervention development have not considered the perspective of patients and their informal carers.

One multidisciplinary approach to address HF and other morbidities is blended collaborative care (BCC), an evolved form of collaborative care (CC). CC is a team-based, patient-centred care strategy (11, 12). Based on Wagner's chronic care model (13), CC is characterized by the involvement of a care manager (CM). The CM, who is a nonphysician health professional (e.g., a nurse), supports patients in coping with everyday life and managing their diseases, e.g., by educating the patients about their conditions, helping to implement evidence-based treatment recommendations, encouraging health behaviours, or monitoring responses to therapy (11, 12, 14, 15).

While the first CC trials focused on improving mental health (16–18), subsequent studies also targeted several outcome measures in single somatic conditions (19–21). In samples of cardiac patients, the CC model mainly showed positive effects on symptoms of depression, anxiety, and quality of life (11, 12, 22, 23). Huffman et al. (24) found a significant improvement in mental health-related quality of life among cardiac patients receiving a CC intervention. A review of CC interventions for patients with heart disease supported their effectiveness in terms of improving mental health (mood symptoms, anxiety), health-related quality of life, and function (25). Although the CC model has been proven effective in patients with one illness, it mostly failed to impact comorbidities (10, 26–28).

To provide better care for multimorbid patients, Katon et al. (29) proposed the “BCC strategy”, an evolution of the CC model that aims to address multiple conditions. In BCC, a CM implements a long-term treatment plan for both somatic and mental conditions in collaboration with an expert team of treatment specialists. BCC is increasingly being implemented in the treatment and care of cardiac patients (9, 30–32). Some studies have confirmed the positive effects of BCC on both mental and physical health (14, 30, 33), whereas others studies have not reported any effects of BCC on somatic conditions. In a recently published RCT, Rollman et al. (9) compared three approaches: a BCC program for treating both HF and depression; a CC program for HF alone; and usual care (see also (31). Over a 12-month period, BCC led to a greater improvement in mood than CC for HF or usual care. However, measures of physical health (e.g., function, rehospitalization, mortality) were not significantly impacted.

One possible explanation for the mixed results regarding the effectiveness of BCC might be that to better deal with the challenge of targeting several conditions and having to prioritize competing treatment plans, the model needs to be further adapted to the needs of multimorbid patients. To date, patients' needs have not been sufficiently considered when creating these treatments (34). Researchers point to the importance of patient involvement in the development of new collaborative models to ensure that they meet patients' needs, to increase adherence and to optimize care (35, 36), especially with respect to multimorbid patients (37). In a study by Kohlmann et al. (38), patients with cardiovascular disorders reported unmet supportive care needs, which indicates that patient-centred care might improve the treatment of cardiac patients.

The patient perspective on BCC for people with multimorbidity and HF has not been systematically studied. Some qualitative studies investigated cardiac patients' perspectives on treatment quality (39), and others retrospectively evaluated CC models for cardiac patients (40, 41) or patients with mental health issues (42–45). However, qualitative research on patients' and carers' needs regarding the development of (B)CC interventions is missing.

An efficient method for communicating patients' needs and deriving appropriate interventions from them is the creation of personas. A persona is a “hypothetical archetype” (46) based on qualitative, quantitative, or mixed data (47, 48). Personas are fictitious characters representing the needs of people in terms of their goals and personal characteristics (46, 49, 50). Although they are imaginary, personas are based on knowledge of real people and are developed as part of an investigative process (50). The development of personas was originally applied in user-centred design studies striving for a deeper understanding of the intended user population of information and communication technologies (48, 51–53). Recently, some studies have used personas in health care research (52, 54–56). In this context, personas mostly serve designers in building electronic health services such as smartphone apps, social media platforms, and telemedicine (48, 57, 58). To our knowledge, qualitative interview data have not yet been used to develop personas representative of potential BCC target persons.

The unsatisfactory results of previous multidisciplinary programs treating patients as a homogenous group suggest that the same type of care for all patients may not address each person equally (Rollman et al. (9). Given that patients have both commonalities and interindividual differences in terms of their care needs and desires, the creation of representative prototype profiles may be a reasonable approach to derive useful recommendations. To gain in-depth insight into the needs of patients, we conducted qualitative interviews. For holistic treatment optimization, the views of informal carers on patients' care were considered as well. Ultimately, increasing knowledge about potential treatment components of a future BCC intervention should lead to outcomes that are more relevant to the target group (59, 60). Thus, the aim of the present study was to investigate the concrete care needs of multimorbid elderly patients with HF and their carers across different countries prior to the development of an international BCC intervention. This study is part of the EU-funded project “Evaluation of a patient-centred biopSychosocial blended collaborative CAre Pathway for the treatment of multimorbid Elderly patients” (61), which aims to develop a BCC intervention and empirically investigate its effects in a randomized controlled trial (RCT).

## 2. Methods

### 2.1. Design

This study employed a qualitative design using patient and carer interviews conducted in Germany, Italy, and Denmark. Patients were enrolled in the study if they were at least 65 years of age and if their medical records included a clinical diagnosis of HF and at least two comorbidities. Data were analysed using framework analysis to create personas. For the interviews, we developed a semistructured interview guide with questions addressing the following topics: education, individual tailoring of treatment plans, monitoring of symptoms, support, coordination, and communication. The topics were derived based on recommendations and previous experience of research experts in the field of multimorbid HF patients and informal carers (31). Each interview topic entailed open questions that generated explorative data (e.g., “What do you think about the way your health status is monitored?”; “What do you think about the support in terms of managing your everyday life?”). Since the interviews were conducted within the framework of an explorative study, no power analysis was carried out. Purposive sampling considering maximum variation was used to select study participants. The study was designed by a diverse team of experienced researchers in the field who have many years of expertise in HF and multimorbidity research. All researchers involved in data collection had comparable qualifications, similar research experience, and no prior relationship with the study participants. For these reasons, the researchers' characteristics were not thought to have a significant impact on the study.

The study procedure was reviewed and approved by the Local Psychological Ethics Committee (LPEK) at the Center for Psychosocial Medicine of the University Medical Center Hamburg-Eppendorf (approved on December 3rd, 2020; LPEK-0237) as well as by the Local Ethics Committee (Comitato Etico di Area Vasta Emilia Centro, CE-AVEC) at the Sant'Orsola-Malpighi Polyclinic, University of Bologna [Azienda Ospedaliero—Universitaria di Bologna, Policlinico S. Orsola-Malpighi] (Protocol N. PG0012699/2021) and by the Research Ethics Committee of the University of Southern Denmark (approved on March 25th, 2021; case no. 21/876). The study is reported according to the EQUATOR standards for reporting qualitative research (SRQR; 62).

### 2.2. Data collection

In total, 42 interviews (25 patients, 17 informal carers) were conducted between May and October 2021 in three different EU countries (Germany, Italy, and Denmark). The semistructured interviews were recorded with an audio recorder and transcribed anonymously. In Germany, patients were recruited through self-help groups, departments of the University Medical Center Hamburg (general practice, cardiology), and other German university hospitals. The interviews either took place on the online platform WebEx or at the Department of Psychosomatic Medicine and Psychotherapy at the University Medical Center Hamburg. In Italy, patients were recruited at the Division of Cardiology, Bellaria Hospital (Bologna), where the in-person interviews also took place. In Denmark, patients were enrolled by their general practitioners (GPs; in the cities Middelfart and Kerteminde), and the interviews were conducted in the participants' homes. The patients decided whether they wanted their informal carers (relatives, life partners, friends, neighbours, etc.) to participate in the study and whether they wanted to be interviewed together or separately. An overview of the study sample can be found in Table 1.

**Table 1:** Overview of the study group (*N* = 42).

Country	Number of patients	Number of carers	Patients interviewed alone	Carers interviewed alone	Patients and carers interviewed together
Germany	5	2	4	1	2 (1 + 1)
Italy	13	10	5	2	16 (8 + 8)
Denmark	7	5	3	1	8 (4 + 4)

### 2.3. Analyses

The qualitative data derived from the open questions were analysed via framework analysis, a case and theme-based approach that reduces data deductively through summarization and synthesis using a matrix, which enables analyses both by case (patient and carer point of view) and by theme (63).

After the interviews were transcribed in the original language, transcripts were examined by highlighting and comparing quotes expressing fulfilled and unfulfilled needs to identify patterns, tendencies, and similarities across participants in each of the countries involved (64). One research team in each country/language analysed the data. Through the process of looking for and clustering similarities and variations in participants’ needs and preferences using matrices, an initial set of seven country-specific personas (two German personas, three Italian personas, and two Danish personas) were created by the researchers in each of the three involved countries independently. The seven personas were both created and translated into English by the local researchers in preparation for the following cross-cultural step of the analyses. Carers were integrated into the descriptions of the personas instead of being constituted in their own persona because carers participated in this study with the patients. Thus, in most cases, patients and carers were interviewed together. In addition, carers were asked about their experiences and opinions regarding the needs and preferences of the patients rather than describing their own needs. Thus, the personas—and the understanding of patients' needs—benefit from entailing both descriptions of themselves by patients along with outside perceptions of them from their carers.

In a second step, the initial personas were deconstructed within a joint two-day meeting of all authors. Across the three countries, clear similarities between some of the personas could be found. These personas were carefully compared, and their common traits were merged, whereas the traits in which they differed were removed. This created a new outline for three cross-country personas. Next, the group of researchers compared the actual participants from the study constituting a new cross-country persona to look for other potentially common traits and patterns between them. Those commonalities were highlighted within each new persona. Finally, important differences between the participants within the personas were reviewed to ensure clarity in the scientific process. Further data comparison and discussion led to the merging and cocreation of the final set of three cross-national personas that are presented in the results section.

The creation of personas was chosen to account for the diversity of patients' and carers' needs, attitudes, and traits while giving them a coherent and connected representation, as the data suggested variation across participants and therefore a “one size fits all” model for a subsequent BCC intervention to be inefficient. While in commercial settings personas are traditionally generated as a byproduct of the investigative process to reach different users and target groups (46, 50) and are given “a name, a life, and a personality” by their designers (64), the personas in this study are exclusively based on the interview data.

## 3. Results

**Figure 1:**
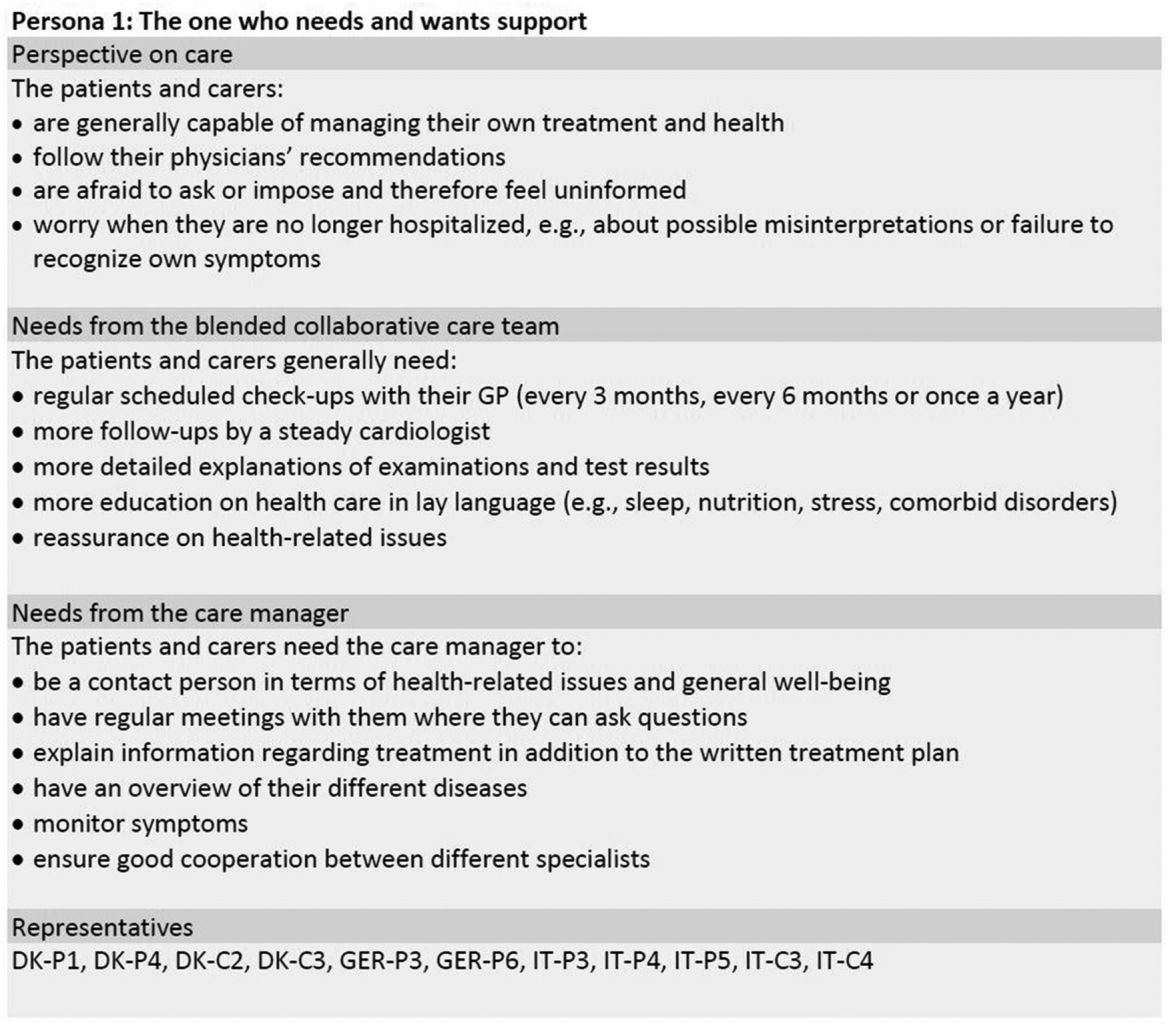
Persona 1 of the final set of personas including patients and informal carers. DK, Denmark; GER, Germany; IT, Italy; C, carer; P, patient.

**Figure 2:**
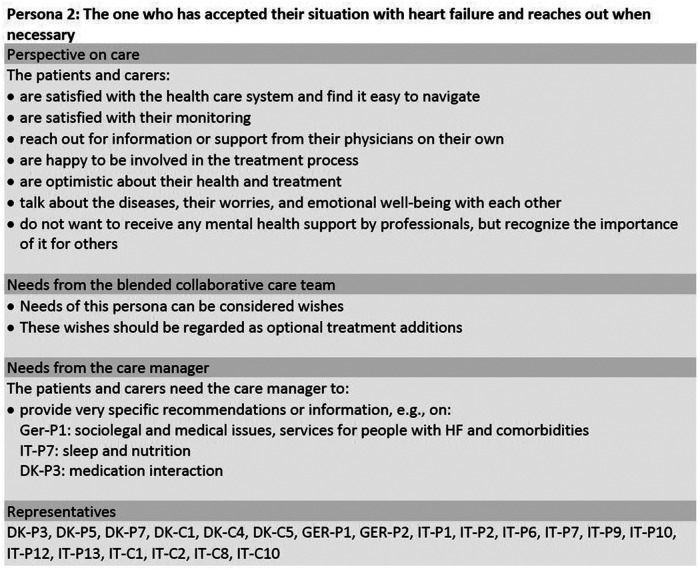
Persona 2 of the final set of personas including patients and informal carers. DK, Denmark; GER, Germany; IT, Italy; C, carer; P, patient.

**Figure 3:**
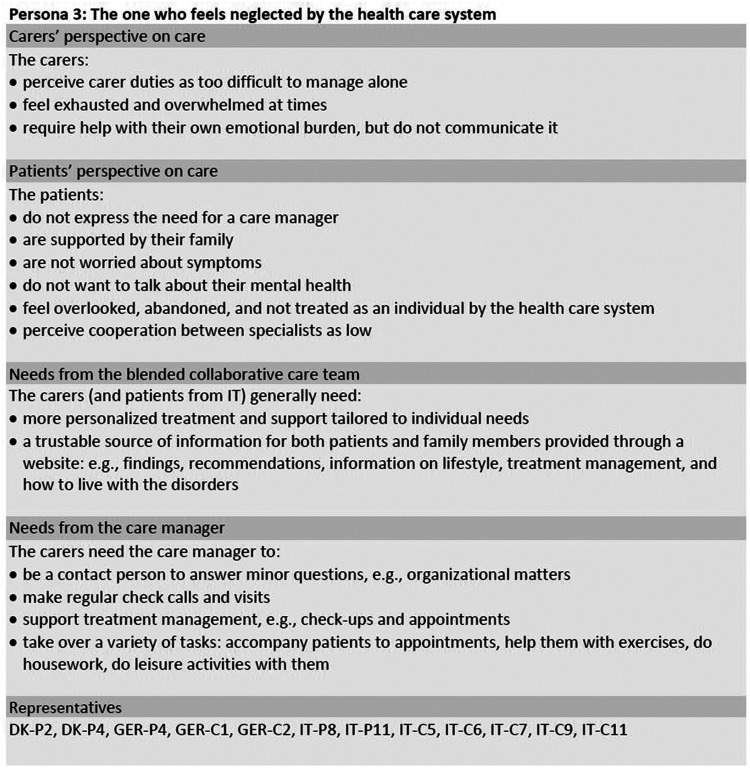
Persona 3 of the final set of personas including patients and informal carers. DK, Denmark; GER, Germany; IT, Italy; C, carer; P, patient.

Framework analysis and merging of the initial seven personas based on interview data of 42 participants resulted in a final set of three personas, which are shown in Figures 1–3. The sample included 25 patients (*n* = 8 females) with a mean age of *M* = 75.76 (*SD* = 7.58) years. Their mean duration of HF was *M* = 15.32 (*SD* = 15.01) years. The sample also included 17 carers (*n* = *12* females) with a mean age of *M* = 65.65 (*SD* = 9.84) years. The three personas are a combination of the initial seven personas, which were entitled “The one who feels overlooked” (DK), “The passive one” (GER), “The one who wants to be supported” (IT), “The one who reaches out” (DK), “The active one” (GER), “The one who is realistic” (IT) and “The one who feels to be left alone” (IT). In the following section, the general characteristics of the participants represented in the three final personas are summarized along with their general needs and in which areas they could benefit from a CM as part of their treatment team. To ensure transparency, areas of differences between the participants in each persona will be explained. In Figures 1–3, the participants to whom the personas refer are indicated via participant codes. Table 2 provides the sociodemographic and clinical characteristics of all patients in the three personas.

**Table 2:** Sociodemographic and clinical characteristics of patients stratified by personas (*n* = 25).

Variable	Persona 1 (*n* = 7)	Persona 2 (*n* = 13)	Persona 3 (*n* = 5)
Age, *M (SD)*	71.86 (4.91)	77.46 (7.88)	75.60 (9.89)
Gender, *n* (%) female	4 (57.1%)	3 (23.1%)	1 (20.0%)
BMI, *M (SD)*	28.84 (5.83)	27.54 (5.16)	28.88 (3.67)
Living alone, *n* (%)	1 (14.3%)	2 (15.4%)	1 (20.0%)
Marital status, *n* (%) married	5 (71.4%)	11 (84.6%)	4 (80.0%)
Years of school education, *M (SD)*	8.17 (4.58)	9.27 (5.16)	8.00 (2.65)
Duration of heart disease (years), *M (SD)*	12.43 (13.50)	18.91 (17.92)	9.00 (6.63)
Number of risk factors, *M (SD)*	3.00 (1.29)	4.09 (1.58)	3.40 (1.14)
Medical conditions			
Heart attack, *n* (%)	3 (42.9%)	5 (38.5%)	4 (80.0%)
Stroke, *n* (%)	1 (14.3%)	1 (7.7%)	0 (0.0%)
Heart valve disease, *n* (%)	2 (28.6%)	5 (38.5%)	0 (0.0%)
Cardiac arrhythmia, *n* (%)	5 (71.4%)	6 (46.2%)	3 (60.0%)
CCS classification, *n* (%) ≥3	0 (0.0%)	1 (7.7%)	0 (0.0%)
NYHA classification, *n* (%) ≥3	3 (42.9%)	4 (30.8%)	3 (60.0%)
Defibrillator, *n* (%)	1 (14.3%)	5 (38.5%)	3 (60.0%)
Bypass surgery, *n* (%)	3 (42.9%)	1 (7.7%)	1 (20.0%)
Recent visit to cardiologist, *n* (%)	4 (57.1%)	7 (53.9%)	4 (80.0%)
Hospitalization for heart disease, *n* (%)	6 (85.7%)	10 (76.9%)	4 (80.0%)

*M*, mean; *SD*, standard deviation; BMI, body mass index; risk factors: smoking, diabetes mellitus, elevated cholesterol, high blood pressure, heart disease in the family; CCS, canadian cardiovascular society; NYHA, New York heart association; recent visit to cardiologist, visit within the last month.

### 3.1. Persona 1: “the one who needs and wants support”

This persona (see Figure 1) is based on eleven participants, seven of whom are females and four males, aged between 61 and 78 years. Persona 1 represents 7 patients and 4 carers from Germany, Italy, and Denmark and is characterized as someone who needs more support. The persona would both like more information about health and lifestyle, and they need support regarding their self-monitoring of symptoms and general health. The latter need ought to be a reassurance as the participants are capable of following recommendations from their GP and of monitoring themselves, but they tend to worry about misinterpreting their own symptoms. The most significant trait to keep in mind with persona 1 is that they need the health care system to reach out to them rather than vice versa.

The participants within persona 1 vary in the amount of time they have been living with HF, their preferences regarding means of communication, and their living situation. In Italy and Denmark, carers and patients were living together, whereas German patients were living alone. In Denmark, participants were all diagnosed with HF within the past 3 years. However, a connection between duration and need for reassurance could not be made across the three countries. Both in Germany and Italy, there was a large variation in the time of diagnosing HF and their current need for support and reassurance.

Additionally, some participants in persona 1 (DK) would like to be part of support groups and to discuss their mental and physical well-being with both peers (e.g., other HF patients) and professionals (e.g., psychologists, therapists, or their GP/cardiologist). Others (GER, IT) would prefer talking to a professional rather than to other patients. While most participants had difficulty talking about the mental distress caused by the disease, only some of the participants (GER, IT) requested support in this area. Furthermore, in Germany and Italy, the participants described a need for assistance with their medications by the CM, whereas the Danish participants needed the CM to help monitor their symptoms.

In general, preferences regarding means of communication varied within and across countries. In Germany and Denmark, the participants saw great value in having a CM to assist the communication with doctors, to give explanations of results, recommendations, and to check on their well-being. Furthermore, they would like to receive written findings, recommendations, and information about specific treatment changes in lay language and short form. Within each country, examples of patients and carers who preferred communicating and getting information on the internet, by phone or email could be found.

### 3.2. Persona 2: “the one who has accepted their situation with HF and reaches out when necessary”

The second persona (see Figure 2) is based on twenty participants, seven of whom are female and thirteen of whom are male, with ages between 54 and 90 years. Persona 2 represents 13 patients and 7 carers from Germany, Italy, and Denmark and can be characterized as someone who has learned to live with their diseases. As this persona is quite content, their requests should be understood as wishes rather than needs. Therefore, these wishes are also very person-specific and should be established together with each patient and carer. The most significant trait of this persona is that they find it easy to navigate the health care system and reach out to it when they need information and support. The participants vary in their specific needs/preferences in terms of whether they want someone to give advice on sleep and nutrition, interactions of different medications or sociolegal issues, for example.

As persona 2 is constituted by the most participants, more variations ought to occur. While all the participants in general felt that their treatment was tailored to their individual needs, some of the participants (DK) worried about the interaction of different medications. They suggested a CM to be someone who has an overview of medications while having expert knowledge on the chemical interaction of their medications. Some of the Italian participants suggested the CM to be someone to remind them of medications and recommendations and with whom they could have more personal contact. In Denmark, most participants within persona 2 had this kind of personal relationship with their GP. The German participants were interested in new research findings and taking part in self-help groups, which contrasted with most of the Danish participants in this persona.

Regarding support for mental health issues, persona 2 in general perceived this area as a private matter. However, they did vary significantly. Whereas some Danish participants by no means wanted to receive any professional support around mental health, others already had conversations about mental health with their GP. Some Italian participants were willing to receive psychological support, while some German participants recommended that a GP (and not a psychologist) should ask empathetic questions about mental well-being. Regarding preferred means of communication, no clear tendency was found within this persona.

### 3.3. Persona 3: “the one who feels neglected by the health care system”

This persona (see Figure 3) is based on twelve participants, among whom seven are female and five are male, aged between 49 and 91 years. Persona 3 consists of 7 carers and 5 patients whose greatest commonality is the feeling of having been neglected by the health care system. Like the other personas presented, it is here also necessary to account for country-specific differences between patients and carers.

Despite feeling neglected, patients identifying with persona 3 saw no need for a CM, which seemed to be due to disappointment with the health care system. In the Italian sample, patients communicated the BCC-specific needs listed in Figure 3. However, patients in the German and Danish samples were above all characterized by resignation regarding their treatment, accompanied by pessimism and scepticism about possible support from health care professionals. In contrast to carers, these patients did not articulate any needs and were reluctant to change their lifestyle.

The Danish patients did not want their carers to be part of the study, which is why “The one who feels neglected by the health care system” does not refer to Danish carers. However, the respective Danish patients made remarks about their carers being worried about them. Therefore, the Danish and German patients within persona 3 may need support according to their carers without the patients wanting it themselves.

Additionally, the German carers belonging to persona 3 indicated that they would like the patients to receive psychological support from a mental health professional, but the patients refused this. One need that was mentioned solely within the Italian sample was support in dealing with complex polypharmacy, drug usage, or injections (e.g., training in insulin injection).

The fact that the description of carers in persona 3 is only based on participants from Germany and Italy might point to a country-specific difference. In Denmark, as opposed to Germany and Italy, carers are rarely expected to be in charge of the general care of a patient (e.g., being responsible for medications, personal care, practical support, etc.). If needed, these areas can be supported by health care staff in the municipalities. Therefore, when carers in Germany and Italy “perceive carer duties as too difficult to manage alone” and “feel exhausted and overwhelmed at times” (Figure 3), this might be a sign of different expectations for carers across countries and the availability of public support.

## 4. Discussion

Although BCC interventions often yield mixed results [e.g. (9)] and researchers emphasize the importance of patient and carer involvement in the development of care models (35, 36), the patient perspective on BCC for multimorbid patients has not been systematically studied. To our knowledge, this is the first international qualitative interview study aimed at exploring patients’ needs and informal carers' perspectives regarding patients' needs in relation to a BCC intervention prior to intervention development and implementation using the creation of prototype profiles, so-called personas. The study was conducted within the international EU-funded project ESCAPE, which will eventually investigate the effects of a BCC intervention through an RCT (61). We investigated the needs of multimorbid elderly patients with HF and their carers regarding health, treatment, and potential CM in three different European countries (Germany, Denmark, and Italy). Framework analysis of 42 interviews with patients and carers in total resulted in three different personas, characterized by different needs, preferences, and attitudes: “The one who needs and wants support”, “The one who has accepted their situation with HF and reaches out when necessary”, and “The one who feels neglected by the health care system”. Whereas carers of the first two personas were content with the patients' care, carers relating to the last persona showed high psychological stress and a need for support regarding their own situation as carers. The location or existence of an informal carer had no impact on patients' affiliation with a specific persona. Compared to the other personas, patients belonging to “The one who has accepted their situation with HF and reaches out when necessary” were older and had a longer duration of heart disease. This observation suggests that a longer duration of illness might lead to more serenity.

A common finding in studies exploring patients' and carers' needs is a lack of disease education and understanding of treatment options. The feeling of not being well informed and involved in treatment decision-making has been found for older and multimorbid patients (65, 66) and those with different chronic conditions, such as patients with chronic kidney disease and their carers (67), patients with chronic obstructive pulmonary disease (COPD) (68), and HF patients and their carers (69, 70). One semistructured interview study with HF patients, for example, stressed patients' knowledge deficit and uncertainty regarding the effectiveness of self-care strategies (71). A systematic review of unmet needs in patients with chronic liver disease highlighted the need for information to understand and manage the disease and awareness and support from health care professionals to better cope with it (72).

In addition to a lack of patient-tailored explanations about diseases and treatments, multimorbid patients reported a lack of a holistic approach (attention to the patients' state of functioning, their limitations in daily life, and their well-being) in a Dutch qualitative study of general practice care needs (73). Accordingly, a recent qualitative review of studies on HF patients' support needs categorized patients' needs into five different themes: self-management, palliative care, supportive care, social support, and continuing person-centred care. The main conclusion of the review was that dynamic and interactive person-centred care was necessary, and a holistic treatment approach was recommended (74).

In a German qualitative study using semistructured interviews on support needs, elderly patients with multimorbidity reported unfulfilled needs regarding emotional management (e.g., coping with loneliness and loss of independence) and social support. Therefore, patients articulated further support from their general practitioners on coping with the disease (75). Similar to this study, HF patients in a German qualitative interview study expressed deficits regarding the quality of individual-tailored information, professional communication and advice, as well as communication and cooperation across health care sectors (76).

Thus, previous research findings are most consistent with the persona “The one who needs and wants support” in this study. For this persona, our study confirms patients' need for education, which is one basic element of BCC (14, 29). In terms of carers' needs in particular, the results of this study regarding “the one who feels neglected by the health care system” are in line with previous studies that identified a high psychological burden in carers of patients with cardiovascular disease (77, 78). A recent review found areas of carers' unmet needs related to insufficient information provision, poor support to manage emotional distress, social isolation, and access to services (79).

Patients such as “The one who feels neglected by the health care system” have hardly been mentioned in the literature thus far. Qualitative research on COPD patients revealed that patients disavowing their needs was a common phenomenon. Despite disavowing their support needs, the COPD patients in a mixed-method population-based longitudinal study by Gardener et al. (80) desired more GP contact than the remaining cohort. In contrast to “The one who feels neglected by the health care system” in our study, subjects indicated no signs of disappointment with the health care system. The authors attributed patients' denial of care needs to stigmatizing beliefs of the sick role.

Although minor country-specific differences were observed, patients and carers in this study showed striking commonalities across countries, which are summarized in three personas. In addition to the benefit of shared decision-making, several conclusions regarding a BCC intervention and the role of a CM can be drawn from these personas. Keeping our findings and personas in mind, we might consider how and to whom more information and education is provided, as different people require different approaches. For Persona 1 (“The one who needs and wants support”), an extra amount of information by checking up on them regularly and allowing time for questions on different occasions is needed. Just providing them with more information after surgery or a regular consultation might leave them feeling just as uninformed as prior to the conversation. Therefore, their need for information provision exceeds the regular education provided in BCC interventions (14, 29), and education should be emphasized in this patient group. While some patients and carers seem to need and welcome support actively initiated by health care professionals such as a CM, for others, a less intense intervention seems to be indicated, as they already feel well cared for overall. One implication of this study is the need to actively pay attention to the emotional stress of carers and formulate relieving support services. Beyond that, there seems to be one type of patient who, as a result of frustrating experiences and disappointment with the health care system, might require a particularly high degree of sensitivity and attention from professionals such as a CM. Therefore, BCC interventions should be targeted to patients' and carers' individual needs in order to derive outcomes relevant to the target group.

Despite its novelty, this study has some limitations. First, participants were not included in the actual design of the BCC intervention. Inviting participants into the design process has, for example, been described in relation to patients suffering from schizophrenia as part of their recovery process by implementing patients' engagement in their own care (81). Such inclusion of patients is called a codesign process, in which technologies are designed with and not for users (81–83). By using codesign, Phillips et al. (84) created personas that “evolved to reveal evidence of shared characteristics and community-wide concerns” of the sample (84). Through the personas, the participants could create distance from their own experiences, which enabled them to, for example, talk more openly about feelings of stigmatization. In similar studies, the approach of involving participants in design and evaluation processes could therefore provide interesting and important insight. Another limitation might be that the personas in our study are based solely on interviews on one occasion. Nevertheless, the personas emerged through an iterative process involving several discussions and revisions by all authors to ensure sufficient foundation. Future studies could benefit from long-term ethnographic observations of participants. As the focus of this study was perceptions of a potential BCC intervention aimed at patients, the interviews did not specifically focus on carers' own support needs.

To our knowledge, this is the first international qualitative interview study on patients' health care needs and informal carers' perspectives regarding patients' needs prior to the development of a BCC intervention. The study used the creation of personas, a user-centred design approach that has only recently been introduced as a method in health care research, to derive intervention recommendations. Three personas (“The one who needs and wants support”, “The one who has accepted their situation with HF and reaches out when necessary”, and “The one who feels neglected by the health care system”) representing health care needs and needs regarding a potential CM of multimorbid elderly patients with HF and their carers were presented. The contrasting personas point to the need for an individualized approach in regard to a BCC intervention. One specific type of patient might need special attention from health care professionals to rebuild trust in health services. To identify the respective personas in individual patients and adjust the treatment approach accordingly, the corresponding needs and traits described could be explored in consultations, e.g., with the help of the presented overviews. The clinical implications of the study include the need to adapt BCC interventions to patients' and carers' needs—for example, by using the personas introduced here. The effects of a customized BCC intervention targeted to patients' and carers' individual needs will be investigated in a forthcoming RCT within the EU-wide project ESCAPE (61). This newly developed biopsychosocial intervention extends research on BCC interventions for patients with comorbidities to a highly vulnerable patient group of older multimorbid patients. The patient-centred, team-based approach, including a targeted treatment plan developed through shared decision-making and enhanced collaborations between patients, their carers, and medical specialists, overcomes the limitations of parallel single-condition care. While carers will be supported in the challenges of their role, patients will receive continuous support for living with the challenges of multimorbidity in terms of self-management and disease coping. Thus, this treatment approach based on the best available evidence and patients' personal preferences, values, and life goals has the potential to make a significant impact on patients' health-related quality of life and lead to improved health outcomes and health care savings.

## Data Availability

The data that support the findings of this study will be available from the corresponding author, PE, upon reasonable request.
